# Effects of mTOR Inhibitors on Components of the Salvador-Warts-Hippo Pathway 

**DOI:** 10.3390/cells1040886

**Published:** 2012-10-19

**Authors:** Jonathan Chiang, Julian A. Martinez-Agosto

**Affiliations:** 1 Department of Human Genetics, David Geffen School of Medicine, University of California, Los Angeles, Los Angeles, CA 90095, USA; Email: jchiang@mednet.ucla.edu; 2 Division of Medical Genetics, Department of Pediatrics, David Geffen School of Medicine, University of California, Los Angeles, Los Angeles, CA 90095, USA

**Keywords:** Hippo pathway, mTOR pathway, Sterile-20 like kinase family, TAZ, TEAD1, signal transduction

## Abstract

The MST/Salvador-Warts-Hippo and mTOR/Akt/PI3K growth signaling pathways have been established as important modulators of cell growth, proliferation and cell survival in controlling organ size in *Drosophila* and mammals. Here, we sought to determine the role of the MST family of kinases, some of which are components of the Hippo pathway, and their closely related Sterile 20-like kinases (STK) as candidates for mediating cross-talk between the Hippo and mTOR pathways. Expression analysis in the HepG2 and MCF7 cell lines demonstrated common expression of MST1/2/4, MAP4K3/4/5, STK 24 (MST3), STK25, STK39, Pak1, SLK, Stradα/β and TAO2. All components of the Hippo signaling pathway are present in both cell lines except for YAP1 in MCF7 cells. mTOR inhibition via rapamycin decreases TAZ levels in HepG2 but not MCF7 cells and increases TEAD1 levels in MCF7 but not HepG2 cells, suggesting a selective role of the mTOR pathway in regulating these Hippo targets in a cell type-specific manner. Furthermore, the cellular localization of TAZ changes in response to mTORC1/2 inhibitors and Akt inhibition. These findings demonstrate the mTOR-dependent regulation of Hippo signaling at the level of the transcriptional regulators TAZ and TEAD1 and highlight the potential role for mTOR inhibitors in regulating Hippo-signaling dependent tumors.

## 1. Introduction

The development of cancer and tumorigenesis in eukaryotic cells occurs as a result of uncontrolled cell growth and division and ultimately leads to abnormal tissue growth. The Salvador-Warts-Hippo (SWH) tumor suppressor and the mammalian target of rapamycin (mTOR) pathways have been implicated as key regulators of both cell proliferation and apoptosis [1]. The SWH pathway, first described in *Drosophila*, is an evolutionarily conserved molecular mechanism that in mammals consists of a cassette of core kinases, including MST1/2 (Hippo homologue), Lats1/2 (Warts homologue), Mob (Mats homologue), as well as an adaptor protein, Sav1 (Salvador homologue) [2]. Earlier studies have shown that the downstream effectors of this kinase cassette include the transcription coactivators YAP1/2 and TAZ (Yorkie homologues), which work in conjunction with the TEAD transcription factor (Scalloped homologue) to promote cell proliferation and survival [3]. Lats acts in association with Mob to negatively regulate these two nuclear proteins by phosphorylation of YAP and TAZ, a sign of active Hippo signaling [4]. In the absence of phosphorylation, YAP and TAZ translocate to the nucleus, where they activate TEAD1 targets [5]. Upstream of the core kinase group, several kinases have also been found to be implicated in the SWH signaling pathway [6]. The mechanisms by which most of these upstream elements are regulated, however, have yet to be elucidated [7,8]. 

Hippo/MST belongs to the family of kinases that share homology with Sterile 20p (Ste20p), a serine/threonine mitogen-activated protein kinase (MAPK) which was first discovered in yeast [9]. Its mammalian homologues, MST1 and MST2, are kinases that comprise a subset of the family of about 28 Ste20-like protein kinases that have been discovered in mammals [10]. These kinases function as messengers for both intracellular and extracellular signals that include signal transduction among various other functions [11]. The Ste20p family consists of two structurally distinct subfamilies, the p21-activated kinase (PAK) family and the germinal center kinase (GCK) family. Ste-20 like kinases have been established to be important regulators of the MAP kinase cascades that respond to extracellular signals to regulate gene expression. One of the MAPK activators, the Ras-ERK pathway, has been found to regulate the mTOR pathway, which uses extracellular signals such as nutrient availability to control cell fate in cell survival, growth and metabolism [12]. This raises the interesting possibility that Ste20-like kinases may regulate or mediate the cross-talk between different growth signaling pathways, in particular MST1 and MST2, which have been identified as major components of the SWH pathway [13]. In this study we examine the expression of SWH pathway components as well as several of the Ste20-like kinases in the hepatocarcinoma HepG2 and breast cancer MCF7 cell lines. We identify a role for mTOR activity in regulating TAZ and TEAD1 levels as well as the intracellular localization of TAZ, linking the growth-promoting effects of the SWH and mTORC1 pathways. 

## 2. Results and Discussion

### 2.1. Hippo Pathway Components and STK Kinases Are Expressed in HepG2 and MCF7 Cell Lines

**Figure 1:**
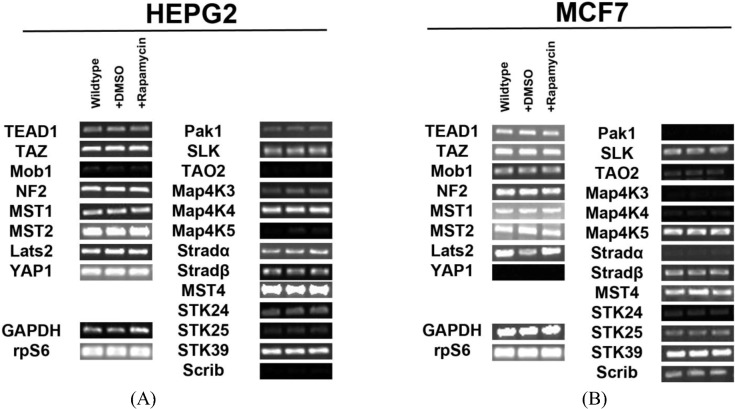
Expression of Hippo pathway components and Ste20-like kinases in HepG2 and MCF7 cell lines. (A) Transcript expression levels of Hippo pathway components (left) and Ste20-like kinases (right) do not change in response to rapamycin treatment in HepG2 cells. Mob1, TAO2, Map4K5 and Scrib are expressed at very low levels. (B) Expression levels of Hippo pathway components and Ste20-like kinases transcripts do not change in response to rapamycin treatment in MCF7 cells. Pak1, TAO2, Map4K3, Map4K4, Stradα and STK24 are expressed at low levels. YAP1 is not expressed. Note: Mob1, Map4K5, STK25 and Scrib are expressed at higher levels in MCF7 cells than in HepG2 cells, while Map4K4 and Stradα are expressed at lower levels in MCF7 cells than in HepG2 cells. For both cell lines, GAPDH and the ribosomal protein rpS6 are included as housekeeping gene controls.

In order to determine the regulation of the SWH pathway in the HepG2 cell line, we first tested for expression of transcripts for the core components of the pathway and Ste20-like kinases. We identified expression of all the components involved in Hippo signaling (Mob1, NF2, MST1/2, Lats2) and their downstream effectors (TEAD1, TAZ, YAP1) in HepG2 cells. We also found that several Ste20-like kinases are expressed in HepG2 cells. These kinases include: Pak1, SLK, TAO2, MAP4K3, MAP4K4, MAP4K5, Stradα/β, MST4, STK24 (MST3), STK25, STK39 and Scrib (Figure 1A). Other genes tested for but not expressed include: Fat2, YSK4 and TAO3. Pak1, TAO2, MAP4K3, MAP4K5, STK24 (MST3), STK25, and Scrib are expressed at low levels.

**Figure S1:**
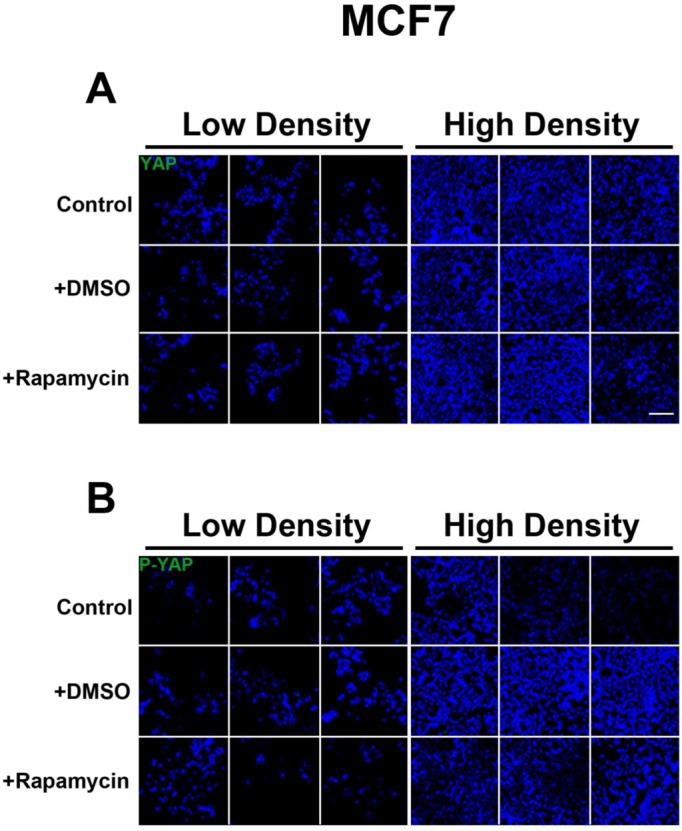
YAP is not expressed in MCF7 cells. (A-B) Immunofluorescent staining of YAP(A, green) and P-YAP (B, green) in MCF7 cells with and without rapamycin treatment at low and high cell density conditions. TOPRO3 (blue) labels nuclei. Scale bars, 100µm.

Similar findings were observed for the MCF7 cell line (Figure 1B). We identified the expression of Hippo signaling components (Mob1, NF2, MST1/2, Lats2) and their targets TEAD1 and TAZ in these cells. Importantly, we noted the absence of YAP transcripts. This result was further confirmed through immunocytochemical analysis (Figure S1). Ste20-like kinases expressed include: Pak1, SLK, TAO2, MAP4K3, MAP4K4, MAP4K5, Stradα/β, STK24 (MST3), MST4, STK25, STK39, and Scrib. Other genes tested for but not expressed include: Fat2, YSK4 and TAO3. Mob1, MAP4K5, STK25 and Scrib are expressed at higher levels in MCF7 cells than in HepG2 cells, while MAP4K4 and Stradα are expressed at lower levels in MCF7 cells than in HepG2 cells. *2.2.Inhibition of mTOR Signaling Does Not Affect Transcription of Hippo and Ste20-like Kinases in HepG2 and MCF7 Cell Lines*

Signaling through mTOR occurs through two different complexes: mTORC1 and mTORC2 [14]. Rapamycin inhibits mTORC1 by inhibiting the interaction of Raptor with mTOR [15,16]. To study the effect of mTOR on Hippo signaling, we blocked mTOR activity by adding rapamycin to HepG2 cells and re-examined expression of Hippo components and Ste20-like kinases. We observed no changes in expression of pathway components and Ste20-like kinases when cells were treated with rapamycin (Figure 1A). Similar studies in MCF7 cells confirmed the same results (Figure 1B). Taken altogether, these results demonstrate that mTOR signaling does not regulate Hippo or Ste20-like kinase levels at the transcriptional level.

### 2.2. Rapamycin Alters TEAD1 Protein Expression in MCF7 Cells

**Figure S2:**
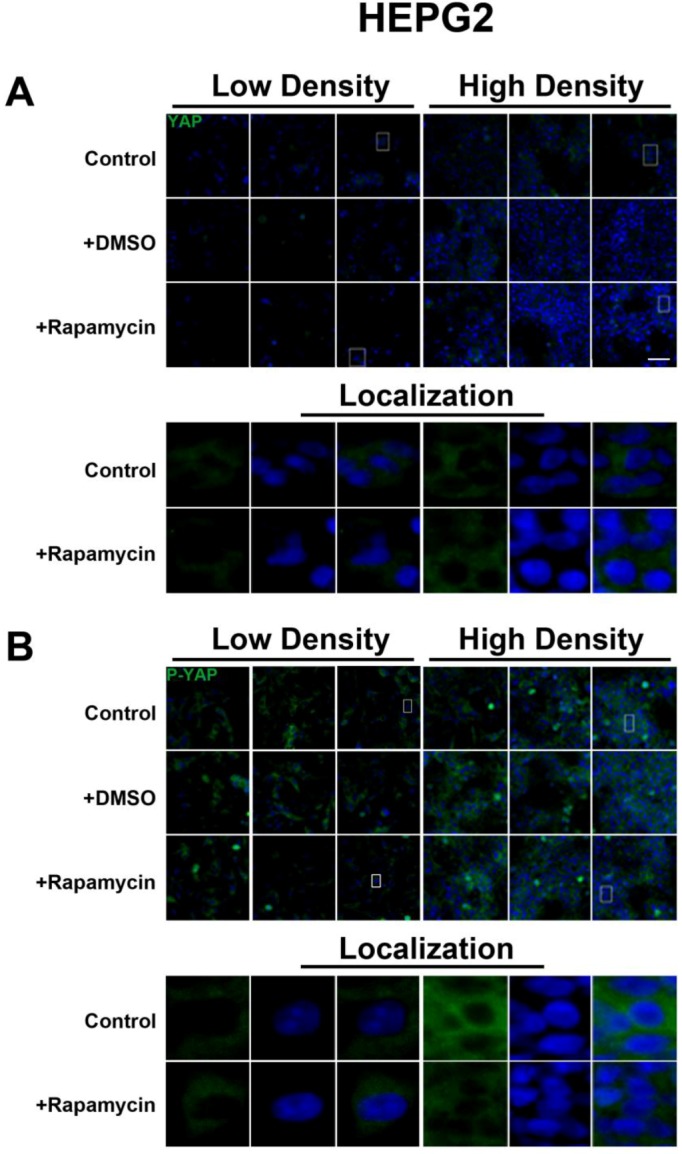
Rapamycin treatment does not affect YAP/P-YAP expression or localization in HepG2 cells. (A-B) Immunofluorescent staining of YAP (A, green) and P-YAP (B, green) in MCF7 cells with and without rapamycin treatment at low and high cell density conditions demonstrates no changes. Bottom panels represent box in top images: localization of YAP or P-YAP at low and high cell density conditions demonstrates cytoplasmic localization and does not change with rapamycin treatment. TOPRO3 (blue) labels nuclei. Scale bars, 50 µm.

We determined the intracellular localization and expression levels of the Hippo target YAP, for both its phosphorylated and unphosphorylated forms, in HepG2 cells, via immunocytochemistry in low and high cell density conditions (Figure S2). We observed that YAP and P-YAP proteins are mainly cytosolic and their expression levels or intracellular localization do not appear to be affected by cell density. Upon treatment with rapamycin, we observed that YAP and P-YAP remained cytosolic with drug treatment and that the expression levels of YAP and P-YAP did not change in response to cell density (Figure S2). 

**Figure 2:**
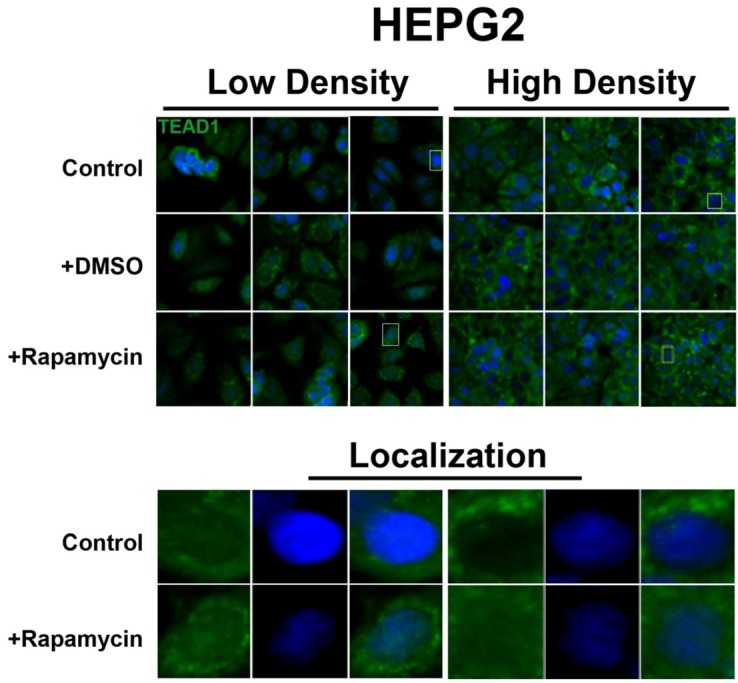
Rapamycin treatment does not affect TEAD1 protein expression levels but affects its nuclear localization in HepG2 cells. TEAD1 (green) expression does not change with and without rapamycin treatment at low and high cell density conditions. Bottom panels represent the boxes in top images: localization of TEAD1 at low and high cell density conditions demonstrates that, in contrast to MCF7 cells, it is mainly nuclear in low density conditions, while it is less nuclear at high density conditions. Rapamycin treatment increases nuclear localization at high density conditions. TOPRO3 (blue) labels nuclei. Scale bars, 20 µm.

**Figure 3:**
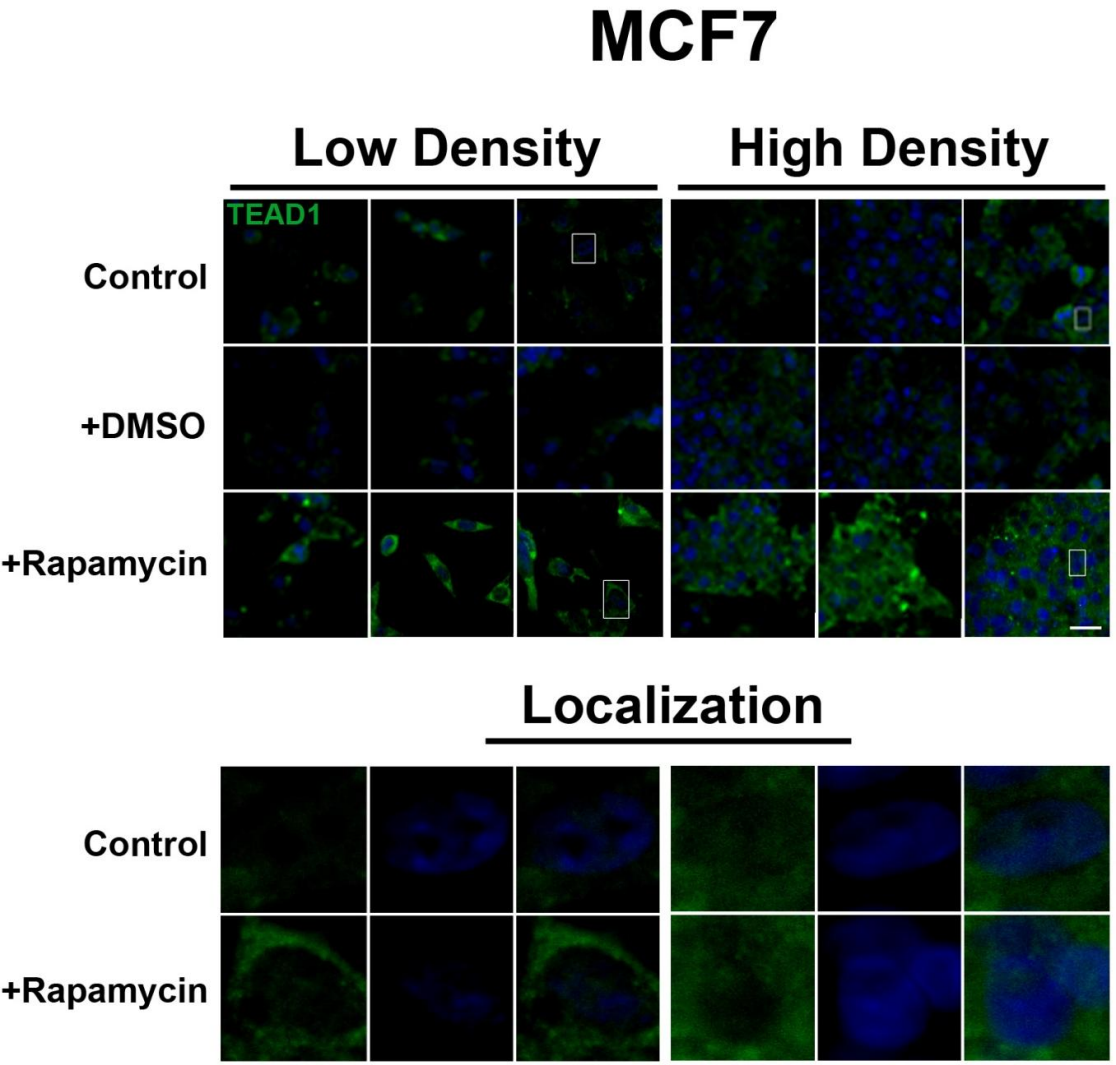
mTOR pathway inhibition increases TEAD1 levels in MCF7 cells. Elevated levels of TEAD1 (green) were observed at both high and low cell densities after treatment with rapamycin. Bottom panels represent the boxes in top images: localization of TEAD1 at low and high cell density conditions demonstrates cytoplasmic localization that does not change with rapamycin treatment. TOPRO3 (blue) labels nuclei. Scale bars, 20µm.

**Figure 4:**
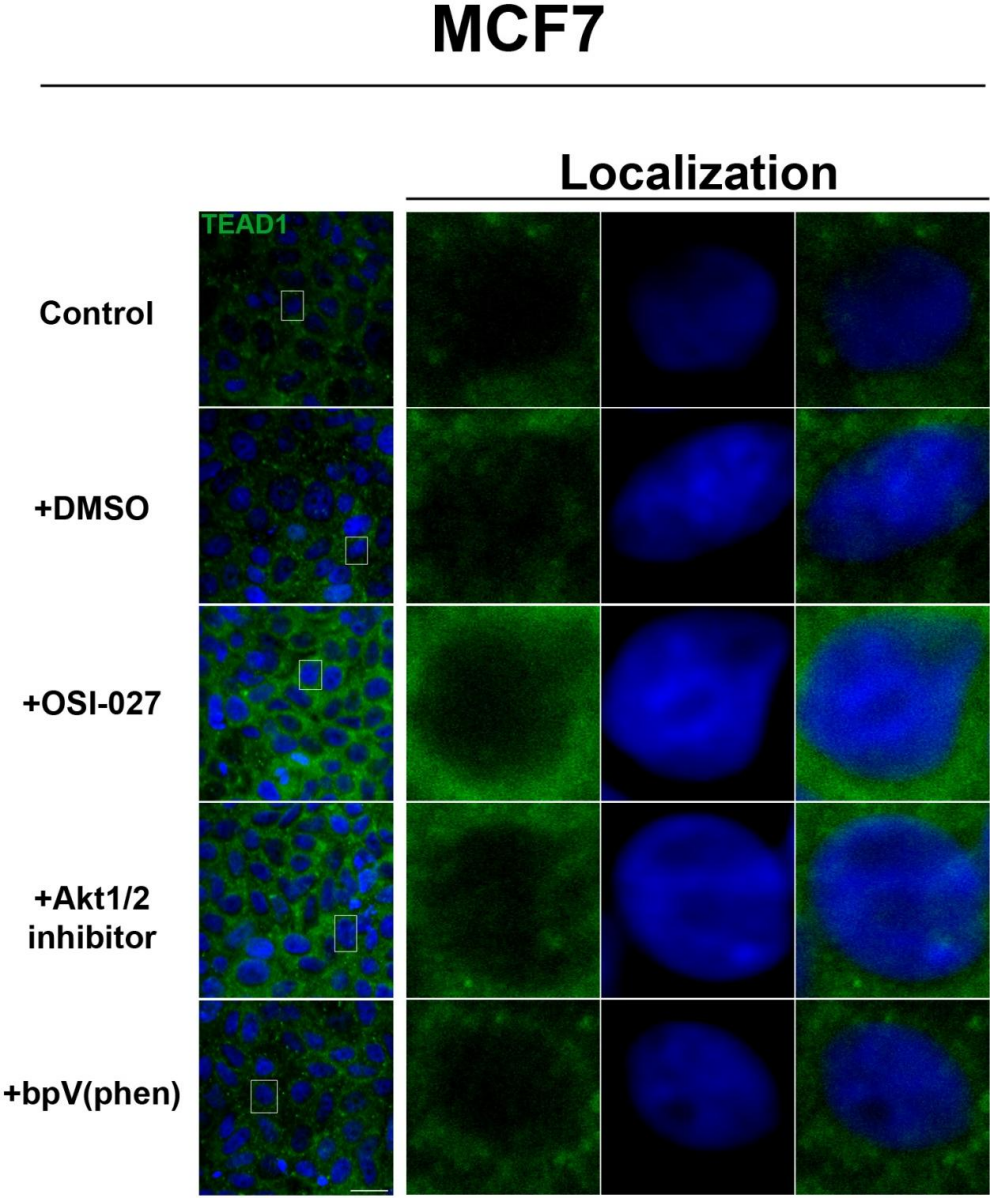
Akt pathway inhibition increases TEAD1 levels in MCF7 cells. Elevated levels of TEAD1 (green) were observed at high cell density conditions after treatment with an Akt inhibitor. The observed effects of rapamycin were confirmed with the mTORC1/2 inhibitor OSI-027. Panels on the right represent boxed images on the left: localization of TEAD1 at high cell density demonstrates cytoplasmic localization that does not change with drug treatment. TOPRO3 (blue) labels nuclei. Scale bars, 20µm.

In contrast, the transcription factor TEAD1, a binding partner for YAP and target of the Hippo pathway, is mainly nuclear at low cell density conditions, but less nuclear at high density in HepG2 cells (Figure 2). While no appreciable change in TEAD1 expression was found upon treatment with drug in both high and low cell density conditions, rapamycin enhances the nuclear localization of TEAD1 in both cell density conditions (Figure 2). However, TEAD1 is mainly cytosolic in MCF7 cells and its expression levels increase upon treatment with rapamycin without affecting intracellular localization (Figure 3). We further confirmed these findings by using an inhibitor of both mTORC1 and mTORC2, OSI-027 [17] (Figure 4). A similar increase in TEAD1 levels was observed upon Akt inhibition, but not with upregulation of Akt activity via treatment with a PTEN inhibitor (Figure 4). These results demonstrate that mTORC1 signaling selectively regulates specific Hippo pathway components and their intracellular localization in a cell-type specific manner: in HepG2 cells, it regulates nuclear localization of TEAD1, while in MCF7 cells, it determines its expression levels.

### 2.3. mTOR Inhibition Affects the Expression Levels of the Hippo Target TAZ in HepG2 Cells But Not MCF7 Cells

**Figure 5:**
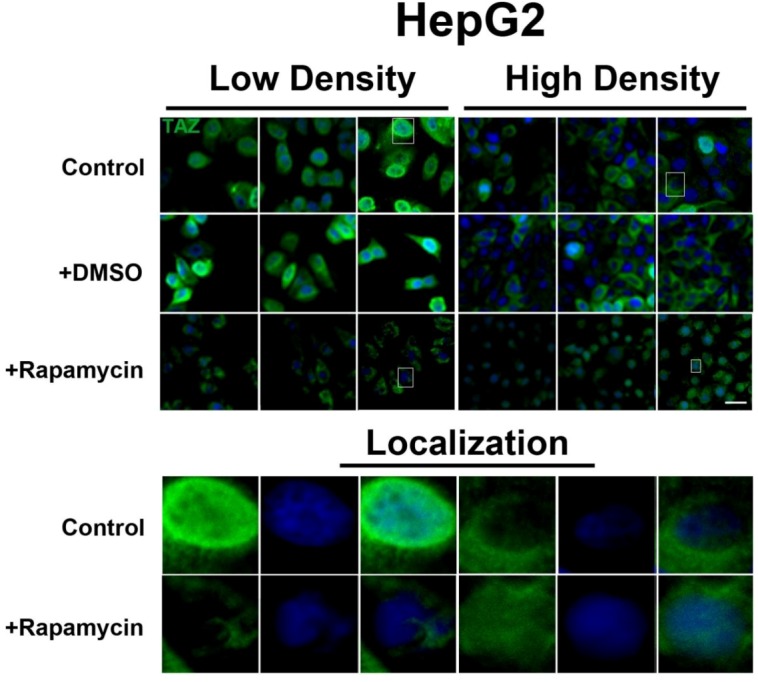
mTOR pathway inhibition affects expression and localization of TAZ in HepG2 cells. Decreased levels of TAZ (green) were observed at both high and low cell densities after treatment with rapamycin. Bottom panels represent the boxes in top images: localization of TAZ at low cell density conditions demonstrates mostly nuclear expression that becomes cytoplasmic upon rapamycin treatment, while in high density conditions, TAZ is mostly cytoplasmic in control cells but shifts to the nucleus upon rapamycin treatment. TOPRO3 (blue) labels nuclei. Scale bars, 20 µm.

**Figure 6:**
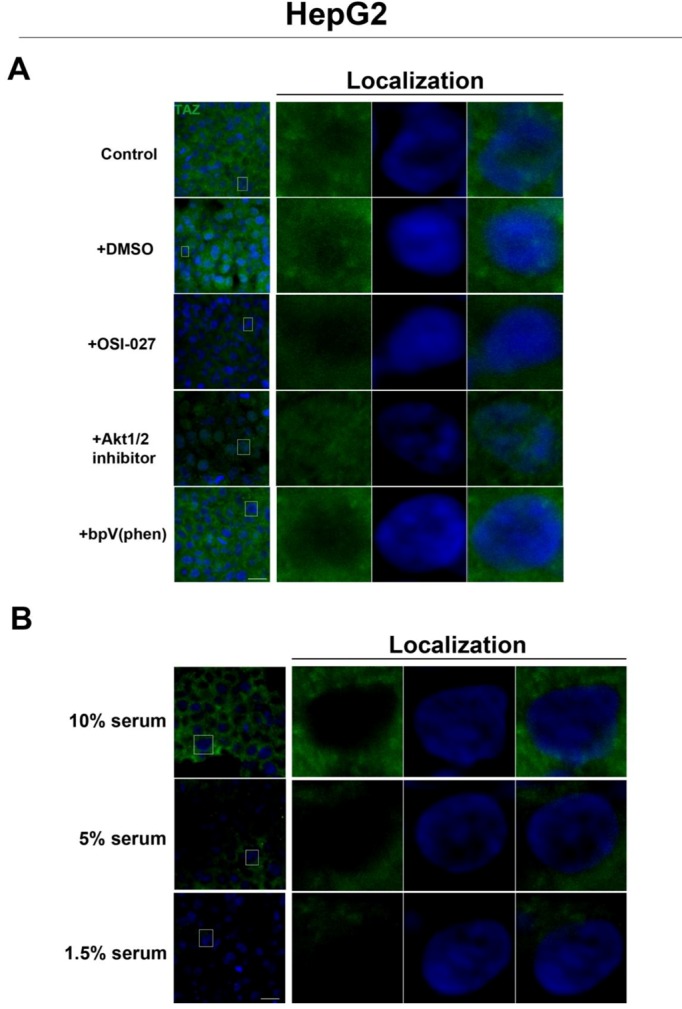
Akt pathway inhibition and changes in serum levels affects expression and localization of TAZ in HepG2 cells. (A) Decreased levels of TAZ (green) were observed at high cell density conditions after treatment with an Akt inhibitor but not with the PTEN inhibitor bpV(phen). (B) Decreased levels of TAZ were also observed at high cell density conditions after a reduction in serum levels. Panels on the right represent boxed images on the left: localization of TAZ in high cell density conditions demonstrates mostly cytoplasmic expression that does not change upon OSI-027 treatment but shifts to the nucleus upon Akt1/2 inhibitor treatment. TOPRO3 (blue) labels nuclei. Scale bars, 20 µm.

**Figure S3:**
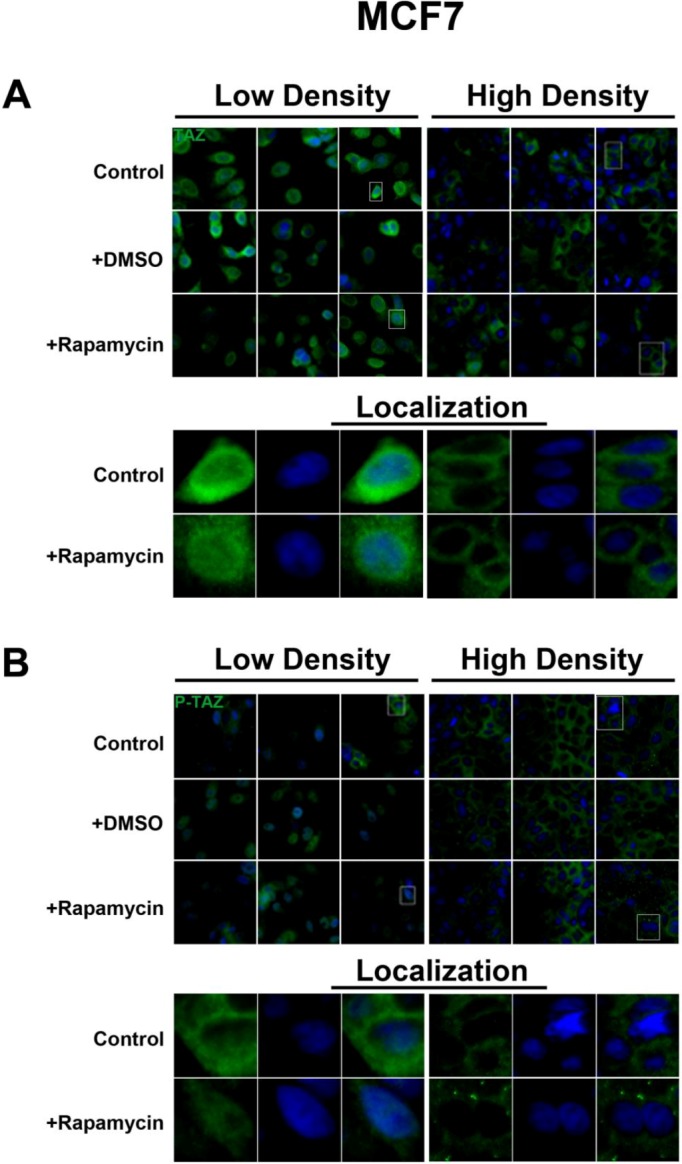
mTOR pathway inhibition does not affect TAZ/P-TAZ expression or localization in MCF7 cells. (A-B) Immunofluorescent staining of TAZ (A, green) and P-TAZ (B, green) in MCF7 cells with and without rapamycin treatment at low and high cell density conditions demonstrates no changes. Bottom panels represent box in top images: localization of TAZ and P-TAZ at low cell density conditions demonstrates mostly nuclear localization that does not change upon rapamycin treatment, while in high density conditions, TAZ and P-TAZ is cytoplasmic and does not change upon rapamycin treatment. TOPRO3 (blue) labels nuclei. Scale bars, 20 µm.

**Figure S4:**
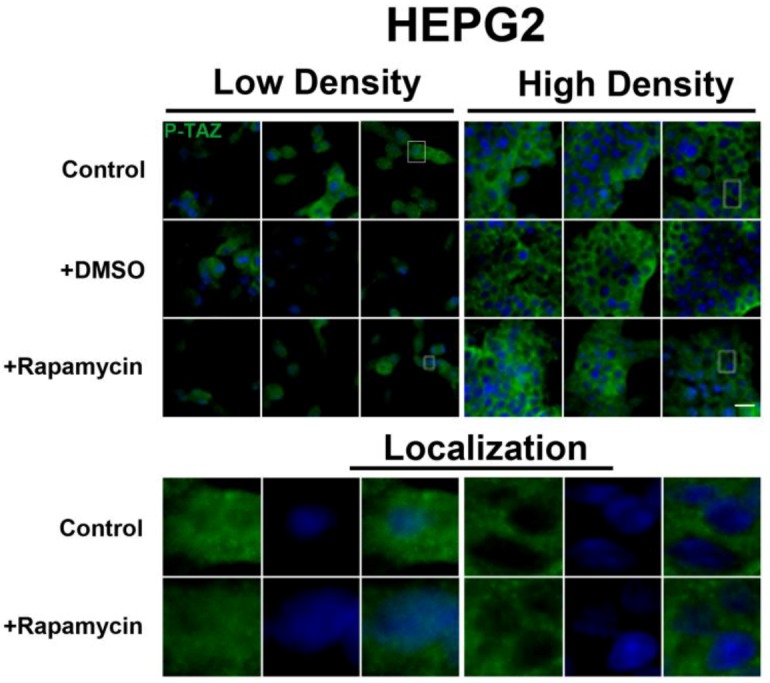
Rapamycin treatment does not affect P-TAZ expression or localization in HepG2 cells. Immunofluorescent staining of P-TAZ (green) in HepG2 cells with and without rapamycin treatment at low and high cell density conditions demonstrates no changes. Bottom panels represent box in top images: localization of P-TAZ at low cell density conditions demonstrates mostly nuclear localization that does not change upon rapamycin treatment, while in high density conditions, P-TAZ is cytoplasmic and does not change upon rapamycin treatment. TOPRO3 (blue) labels nuclei. Scale bars, 20 µm.

To further investigate the relationship between the Hippo and mTOR pathways, we next examined the effect of mTOR inhibition on the Hippo target TAZ. In both cell lines, TAZ and P-TAZ are localized in the nucleus at low cell density, but translocate to the cytosol in response to increased cell density (Figure 5, Figure S3, Figure S4). However, inhibition of mTOR signaling via rapamycin treatment yielded no change in localization or expression of TAZ or P-TAZ in MCF7 cells (Figure S3). In contrast, we found that inhibition of the mTOR pathway via rapamycin treatment in HepG2 cells resulted in decreased expression levels of TAZ without affecting levels of P-TAZ (Figure 5 and Figure S4). Additionally, TAZ in rapamycin-treated HepG2 cells at low cell density appears to translocate to the cytosol (Figure 5), while TAZ in rapamycin-treated cells at high density appears to translocate to the nucleus, compared to controls (Figure 5). Interestingly, while inhibition of Akt decreases TAZ levels and causes an increase in its nuclear localization as is the case with rapamycin (mTORC1 inhibition with partial mTORC2 inhibition), to our surprise OSI-027 (mTORC1 + mTORC2 inhibition) treatment decreases TAZ levels but does not increase nuclear localization (Figure 6A). A similar decrease in TAZ levels occurs upon reduction of serum levels (Figure 6B). These results indicate that mTORC1 signaling regulates levels of TAZ, while its localization to the nucleus may be more dependent on mTORC2 in high cell density conditions. 

### 2.4. Discussion

Previous studies have shown that the SWH signaling pathway is critical in regulating cellular development and homeostasis [2,7], and interference with pathway components results directly in dysregulation of tissue and organ size. Our results confirmed expression of the key SWH pathway machinery (Mob1, MST2, Lats2) transcripts in HepG2 and MCF7 cells. We also confirmed the transcriptional expression of several Ste20p-like kinases in HepG2 and MCF7 cells. Upon treatment with rapamycin, the transcript expression of both SWH pathway components and Ste20p-like kinases in these cell lines did not change, demonstrating that mTOR does not regulate these elements of the Hippo pathway at the transcriptional level. This data also suggests that select Ste20p-like kinases (Pak1, SLK, TAO2, MAP4K3, MAP4K4, MAP4K5, Stradα/β, MST4, STK24, STK25, STK39, and Scrib) are not regulated by mTORC1 signaling. Despite the lack of effect on transcription levels, the known role of mTOR in promoting protein translation suggested the possibility that it may regulate components of the Hippo pathway at the translational level. 

TEA Domain family member 1 (TEAD1) is a known homologue of the *Drosophila melanogaster* transcription factor Scalloped, a known effector of the Hippo pathway that interacts with WW-domain coactivators. In vertebrates, these coactivators are encoded by the *YAP* and *TAZ* genes and they promote cell cycle progression [18], cell proliferation, and differentiation [19]. TEAD1 has been shown to play a role in retaining TAZ in the nucleus to promote cell proliferation [20], mediate YAP-dependent growth control [21], and its knockdown has been shown to decrease cell proliferation [22]. While increases in TEAD1 expression levels are associated with decreased survival in prostate cancer [22], and in conjunction with TAZ induces epithelial-mesenchymal transition [3], there is limited data on the role of TEAD1 in cancer. Our immunocytochemical results demonstrate a differential effect of rapamycin on increasing TEAD1 levels in MCF7 but not HepG2 cells, and this was confirmed with the mTORC1/2 inhibitor OSI-027. This difference is likely due to a cell-type specific effect of TEAD1 in breast cancer cells that awaits further study. 

The lack of changes in P-YAP in response to rapamycin treatment suggests that there is no YAP-mediated interaction between the SWH and mTOR pathways both at the transcriptional and post-translational levels. However, our study was limited to the use of an antibody that recognizes phosphorylation at the Serine 127 and Serine 89 residues [23]. This accounts for the higher levels of P-YAP compared to total YAP seen in HepG2 cells. Therefore, it remains to be determined if additional phosphorylation sites may potentially mediate cross-talk between the two pathways. 

TAZ is a demonstrated transcriptional coactivator regulated by the Hippo pathway that promotes cell proliferation and epithelial-mesenchymal transition [23,24]. Phosphorylation at key residues by Hippo pathway kinases causes retention of TAZ in the cytoplasm and prevents its growth-promoting activity [22]. We did not observe changes in P-TAZ levels or intracellular localization upon rapamycin treatment in both cell lines. TAZ can be phosphorylated at four different serine residues (Ser89, Ser66, Ser117, Ser311) [22]. We examined only the Ser89 residue because it is most commonly dependent on Hippo signaling. It is possible that the expression levels of TAZ that has been phosphorylated at other sites in the protein change in response to rapamycin treatment. More importantly, our results demonstrate that the intracellular localization of TAZ is actively regulated in both MCF7 and HepG2 cell lines. In conditions of high cell density, TAZ translocates to the cytosol and this is associated with contact-inhibition of cell growth. However, inhibition of mTOR signaling via rapamycin had no effect in MCF7 cells but decreased TAZ levels in HepG2 cells, as do decreases in serum levels. This decrease in expression was accompanied by TAZ translocation to the nucleus at high density, likely the result of inhibition of translation of proteins required for its cytosolic retention. Similarly, Akt inhibition decreases its expression levels and promotes its nuclear localization, consistent with the changes observed with rapamycin. Interestingly, while mTORC1 inhibition with rapamycin in high cell density conditions promotes the nuclear localization of TAZ, the mTORC1/2 inhibitor OSI-027 has no effect on localization in HepG2 cells. This suggests a potential role for mTORC2 in the nuclear localization of TAZ upon mTOR inhibition. 

Based on our findings, TAZ represents a novel biomarker for investigation in liver cancer, and its responsiveness to rapamycin specifically in this type of tumor suggests a potential therapeutic intervention for hepatic tumors in which TAZ is upregulated. Elevated levels of TAZ have been correlated with breast cancer cell migration, invasion, and tumorigenesis, while TAZ knockdown in breast cancer cell lines have impaired the tumorigenic ability of those cells [25]. These findings have defined TAZ as an important regulator of breast cancer development and progression. However, the correlation of TAZ levels with other types of tumors has been limited to a few cases, including non-small cell lung cancer cell lines [26] and papillary thyroid carcinoma [27]. The decrease in TAZ levels upon rapamycin treatment suggests a potential role for the mTOR pathway in maintaining TAZ levels, and further studies of this mechanism in other cancers may help elucidate the role of Hippo signaling in tumorigenesis. 

## 3. Experimental Section

### 3.1. Cell Lines and Cell Culture

**Figure S5:**
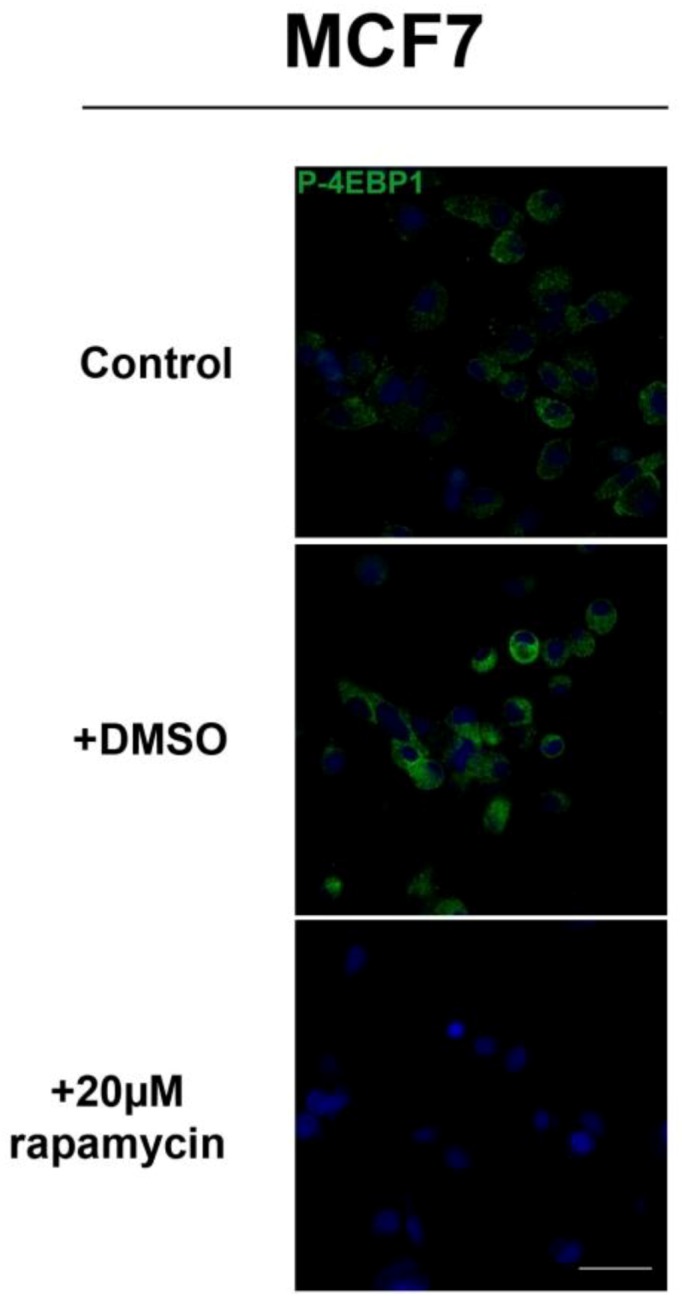
Rapamycin treatment decreases P-4EBP1 levels. Immunocytochemistry for the mTORC1 target P-4EBP1 (green) demonstrates a decrease in levels upon treatment with rapamycin. TOPRO3 (blue) labels nuclei. Scale bars, 20 µm.

HepG2 and MCF7 cells (ATCC, Manassas) were cultured in Dulbecco’s Modification of Eagle’s Medium (4.5 g/L glucose, with L-glutamine and sodium pyruvate; Cellgro, Manassas) and supplemented with 10% heat-inactivated fetal bovine serum (Life Technologies, Grand Island) and Penicillin-Streptomycin-Neomycin antibiotic mixture (Life Technologies, Grand Island) and grown at 37 °C and 5% CO_2_. For experiments, cells were plated on 60 mm petri dishes and grown for 48 hours to 70%–80% confluence before drug treatment. Petri dishes were separated into triplicates, with each dish exposed to one of three conditions: (1) No drug treatment (control) (2) 0.1% DMSO (Fisher Scientific, Fair Lawn) or (3) 20 nΜ rapamycin in 0.1% DMSO (LC Laboratories, Woburn) and incubated for 24 hours. Rapamycin effectiveness was determined by effects on P-4EBP1 immunofluorescence (Figure S5). Other drugs used in our study include: 10 nM Akt1/2 kinase inhibitor (Sigma-Aldrich, St. Louis), 100 nM bpV(phen) (Calbiochem, Billerica), and 500 pM OSI-027 (ChemieTek, Indianapolis).

### 3.2. RNA Extraction, Purification and cDNA Synthesis

Triplicate dishes of drug-treated cells were lysed using TRIzol® Reagent and processed as per manufacturer's protocol. RNA was then treated with DNase I (Roche, Indianapolis) for 30 minutes. RNA was extracted with Phenol/Chloroform/Isoamyl Alcohol (Fisher Scientific, Fair Lawn), 3M Sodium Acetate Trihydrate (Sigma, St. Louis), 1 mM EDTA, glycogen (Ambion, Austin) and ethanol. cDNA was synthesized using the Superscript® III First-Strand Synthesis System for RT-PCR (Life Technologies, Carlsbad). For each condition, 1 µg of DNAse-treated RNA was used. 

### 3.3. Semi-Quantitative PCR Amplification of Target Genes

**Figure S6:**
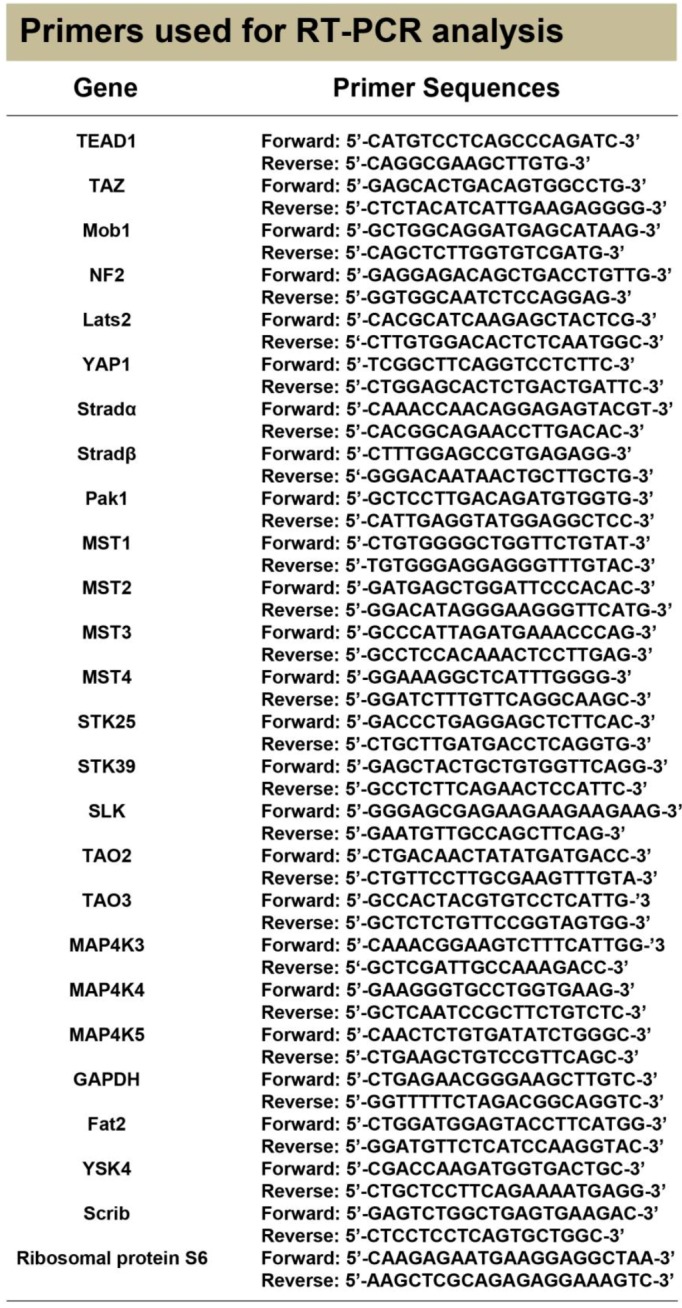
Nucleotide sequences of PCR primers used in the study.

PCR primers (Figure S6) were designed to span introns to ensure specificity of amplification. A 100ng sample of cDNA was subjected to PCR using the following procedures: 94 °C for 3 minutes, followed by 34 cycles of 94 °C for 45 seconds, 55 °C for 30 seconds, and 72 °C for 90 seconds. Samples were then incubated at 72° for 10 minutes. All cDNA synthesis and PCR procedures were performed using the Bio-Rad C1000™ Thermal Cycler. PCR products were resolved in a 1% agarose gel. Each experimental condition and gene expression level was replicated in a minimum of four samples. 

### 3.4. Immunocytochemistry

Primary antibodies used in our study included: TEAD1 (1:100; Lifespan Biosciences, Seattle), TAZ, P-TAZ (1:200; Ser89, Santa Cruz Biotechnology, Santa Cruz), P-4EBP1 (1:400), YAP, and P-YAP (1:100; Ser127, Cell Signaling Technology, Danvers). Cells were plated on poly-D-Lysine coated coverslips (BD Biosciences, Bedford) and grown to 80% confluence, washed once with PBS (Cellgro, Manassas), and fixed with 4% paraformaldehyde. Cells were washed three times with PBS, permeabilized with 0.05% Triton X-100/PBS and blocked with 10% NGS/PBS. Cells were then incubated overnight at 4 °C with primary antibody, rinsed twice with 10% NGS in 0.05% Triton X-100/PBS and incubated for two hours with secondary antibody coupled to FITC (Jackson ImmunoResearch Laboratories, West Grove) in a 1:400 dilution. Finally, coverslips were washed twice with PBS, incubated in TOPRO3 (Life Technologies, Eugene) with a 1:500 dilution and washed once with water. Coverslips were then mounted onto microscope slides using Vectashield (Vector Laboratories, Burlingame) mounting medium and examined using a Carl Zeiss LSM 310 Laser Scanning Confocal Microscope. Each of the experimental findings was replicated with a minimum of five samples. 

## 4. Conclusions

**Figure 7:**
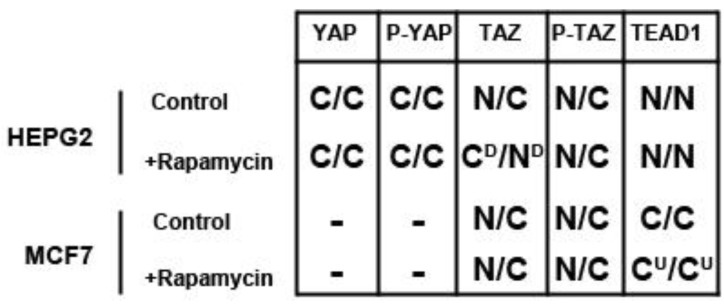
Effects of rapamycin treatment on Hippo pathway components in HepG2 and MCF7 cells. Cytoplasmic (C) or nuclear (N) localization and downregulation (D) or upregulation (U) of Hippo pathway components at low density (left) and high density (right) conditions.

In this study we demonstrate expression of all components of the Hippo signaling pathway in both the HepG2 liver and MCF7 breast cancer cell lines, except YAP in MCF7 cells. mTOR inhibition via rapamycin treatment decreases TAZ levels in HepG2 but not MCF7 cells and increases TEAD1 levels in MCF7 but not HepG2 cells, while altering the nuclear localization of TAZ depending on the conditions of cell density (Figure 7). Akt inhibition causes similar effects to mTOR, increasing TEAD1 in MCF7 cells and decreasing TAZ levels in HepG2 cells, but causing nuclear accumulation of TAZ. Our findings suggest a novel selective role of the mTOR pathway in regulating these Hippo targets in a cell type-specific manner and highlight the potential role for mTOR inhibitors in regulating Hippo-signaling dependent tumors.
